# Associations between extracurricular arts activities, school-based arts engagement, and subsequent externalising behaviours in the Early Childhood Longitudinal Study

**DOI:** 10.1038/s41598-023-39925-6

**Published:** 2023-08-24

**Authors:** Meg E. Fluharty, Jessica K. Bone, Feifei Bu, Jill K. Sonke, Daisy Fancourt, Elise Paul

**Affiliations:** 1 Research Department of Behavioural Science and Health, Institute of Epidemiology & Health Care, University College London, 1-19 Torrington Place, London, WC1E 7HB UK; 2 Center for Arts in Medicine, University of Florida, Gainesville, FL USA

**Keywords:** Human behaviour, Psychology

## Abstract

Adolescent externalising behaviours are associated with numerous long-term negative outcomes, although most research is intervention-based as opposed to risk reduction. Arts engagement has been associated with numerous beneficial factors linked to externalising behaviours, yet direct evidence linking them in longitudinal studies is lacking. Data from the Early Childhood Longitudinal Study were used, with baseline at 5th grade and outcomes measured at 8th grade. Ordinary least squares (OLS) regression was used to examine individual-level associations between extracurricular and school-based arts engagement with externalising behaviours. OLS regression was also used to examine associations between school-level arts classes and facilities with an administrator-reported index of externalising behaviours in the school. All models were adjusted for sociodemographic factors. Individual-level analyses were clustered by school. At the individual level, engaging in a greater number of extracurricular arts activities was associated with fewer externalising behaviours, although there was no association for school-based arts engagement. There were no school-level associations between arts classes or adequate arts facilities and externalising behaviours. Our results suggest extracurricular arts activities may be beneficial in reducing the risk for externalising behaviours, but the relationship is seen at an individual-level of engagement rather than based on school-level provision or facilities.

## Introduction

Externalising behaviours such as conduct problems and hyperactivity/inattention are common during adolescence. Adolescents aged 13–18 have a 4% lifetime prevalence of Attention Deficit Hyperactivity Disorder (ADHD), and 6% lifetime prevalence of conduct disorder^1^. Externalising behaviours are associated with a number of long-term negative outcomes at the individual level including leaving school without qualifications^2^, more severe symptoms of depression and anxiety in middle age^3^, lower rates of workforce participation in adulthood^4^, adult criminality^3^, substance use disorders^2^, and increased mortality rates^5^. In addition to the individual-level impacts of externalising behaviours, these behaviours may also influence others within the school environment. Those experiencing peer victimisation in adolescence also experience poorer health, social, and economic outcomes in adulthood^6^. While schools are intended to be a safe space for students, in the US in 2019 nearly 1 in 5 high school students were bullied on school property, 8% were in a physical fight, and 7% threatened or injured with a weapon, while 9% stayed home from school because they felt unsafe^7^.

The vast majority of the literature on predictors of externalising behaviours has focused on risk factors. Pre-natal influences (e.g. maternal smoking), negative family environment, and genetics have all been found to increase the risk of externalising behaviours^8^. Aspects of the school environment such as class size, socioeconomic area^9^, and negative teacher–child relationships^10^ may also contribute to the initiation or exacerbation of externalising behaviours. A number of personal characteristics may underly externalising behaviours including deficits in reward function^8^, negative self-esteem^11^, poor coping skills^12^, impulsivity^13^, and emotion dysregulation^14^. Although there are a number of risk reduction factors such as social competence^15^, resilience^16^, and adaptive emotional regulation strategies^17^ associated with externalising behaviours, less research has focused on these areas. One group of activities known to be associated with each of these protective factors is arts engagement^18^. Arts engagement can take many forms, from painting, to making music, to going to museums, and these varied activities may have a range of benefits.

There is evidence that arts engagement can support a number of outcomes including increased social interaction and cohesion, provision of supportive coping skills, improved emotion regulation, decreased mental distress^18^, better executive functioning^19^, and enhanced socioemotional development^20^. Many of these beneficial outcomes are related to the problems experienced by those with externalising disorders. Previous evidence suggests that art therapy (whereby arts activities are delivered by a trained therapist) can be used as an effective intervention for externalising behaviours^21^. However, there is a lack of resources to provide therapy interventions to all those who could benefit from them^22^. Further, individuals who could benefit from these services are only identified if the problem behaviours are reported^22^**.** Due to these shortcomings, it is relevant to examine whether broader arts engagement could reduce the risk of externalising behaviours occurring in the first instance. There is some evidence to suggest creativity and artistic ability are helpful in reducing the risk of externalising behaviours^23–28^, but the literature is limited. Therefore, more work is needed to identify whether arts engagement could be a positive strategy in preventing or reducing externalising behaviours during adolescence.

It is also relevant to explore whether engaging in the arts either as part of school curriculum or as an extracurricular activity has a bearing on any relationship with externalising behaviours. The 2015 Obama era US education legislation 'Every Student Succeeds Act' (ESSA) reduced the previous strict federal oversight of education and increased local flexibility in use of funds^29^. Consequently, the introduction of ESSA provided more flexibility within the curriculum: arts (e.g. visual arts, music, drama, dance) may be included as part of a 'well-rounded education' in schools^30^. But whether the arts are included varies substantially from school to school as well as by state and district^29^. Therefore, some students have extensive arts available as a part of their curriculum, while others may have none. This inclusion of the arts within the curriculum is important as research has shown that when arts activities are provided within schools, engagement patterns are similar across demographic and socioeconomic groups^31^. However, when children engage in the arts as part of extracurricular activities, arts engagement is socially and geographically patterned and linked with structural opportunities and barriers, with those from lower socioeconomic backgrounds less likely to engage with the arts^31^. Whilst this research has come from outside the US, concerns about unequal access to extracurricular arts activities has been voiced in the US too^32–36^. Schools therefore have an important role in providing universal access to the arts. But whether there are differential associations between externalising behaviours and engagement as part of school curriculum or as an extracurricular activity needs to be explored further.

Therefore, the current study uses a large nationally representative cohort of children progressing from kindergarten through to 8th grade in the US (the Early Childhood Longitudinal Study [ECLS]) to explore whether extracurricular and school-based arts engagement are associated with externalising behaviours in adolescence. We examined individual-level associations between extracurricular art activities and school-based arts availability with parent-reported externalising behaviours, as well as school-level associations between adequacy of arts facilities and administrator-reported externalising behaviours. We hypothesised that increased arts engagement in 5th grade would be associated with reduced likelihood of externalising behaviours in 8th grade at both individual and school level.

## Methods

### Participants

Participants were drawn from the Early Childhood Longitudinal Study (ECLS)^37^, a nationally representative study of children, their parents, teachers, and school administrators from kindergarten (1998–1999) through the end of middle school (8th grade; 2007) in the United States. Data were collected on the children’s cognitive, social, emotional, and physical development as well as the home environment, home educational activities, school environment, classroom environment, classroom curriculum, and teacher qualifications^37^. We used data collected from parents, teachers, and school administrators in the study participants’ 5th and 8th grades.

For the individual-level analyses, we restricted our sample to parents who participated in both the 5th and 8th grade and had non-missing data on outcome variables in 8th grade. A total of 8610 parents participated in the 8th grade wave. Of these, 8315 also participated in the 5th grade wave and had non-missing data on our outcome measures, forming our final analytical sample (see Supplementary Table S1 for details on pattern of missingness in study variables).

The sample for school-level analyses was restricted to schools that participated in the 5th and 8th grade waves and had non-missing outcome variables in 8th grade. A total of 357 schools participated in 5th and 8th grades and had non-missing data on our outcome measures.

### Ethical considerations

All methods were performed in accordance with the relevant guidelines and regulations. Written parental informed consent was obtained for all participant inclusion in ECLS^37^. The analysis was approved by the UCL Research Ethics Committee [18839/001] and University of Florida Institutional Review Board [IRB01901792].

### Outcome variables

At the individual level, externalising behaviours were measured by parental report of the Strengths and Difficulties Questionnaire (SDQ)^38^ when children were in 8th grade. The SDQ is a 25-item questionnaire measuring prosocial behaviour and psychopathology in children and young people between the ages of 3–17. It includes five subscales (emotional symptoms, conduct problems, hyperactivity/inattention, peer relationship problems, and prosocial behaviour). Response options are ‘not true’, ‘somewhat true’, and ‘certainly true’. We used the summed scores of the conduct problems and hyperactivity/inattention subscales to create a continuous score (0–20), with higher values indicating more externalising behaviours^38^.

School-level outcomes were collected in 8th grade from school administrator reports of externalising behaviours^37^. Administrators were asked to report the frequency of 6 externalising behaviours [class cutting, physical confrontations, theft, vandalism, bullying, and classroom disorder] occurring in the school, with responses on a five-point scale ranging from ‘never’, to 'occasionally', 'once a month', 'once a week', and ‘daily’. Responses were summed to create an externalising behaviours index (total possible range 0–24), with higher scores indicating more externalising behaviours.

### Predictor variables

For individual-level analyses, the extracurricular arts activities variable was derived from parental reports of their children’s extracurricular arts activities in 5th grade. Parents were asked: ‘In the past year, has your child participated in [dance lessons, music lessons, art classes or lessons, organised performing art programmes]?’, and were required to check either yes or no for each of the four activities. Responses were then summed to create a continuous score (0–4) of the number of extracurricular arts activities the child had engaged in during the past year.

Two school-based arts engagement variables were examined in both the individual-level and school-level analyses. First, the arts classes variable was derived from questions asked of each child’s teacher: ‘How often do children in your class work on lessons or projects in the following areas [music, musical instruments, art, or art materials]?’ and ‘How often do children in your class use computers for [music composition, art]?’. Responses were made on 5- to 7-point scales, with frequencies ranging from ‘never’ to ‘daily’. Each item was dichotomised to indicate ‘less than once a week or never’ vs '1–2 times a week or more' and summed to create the total number of arts classes offered at least weekly (0–6). This approach gives an indication of the diversity of arts engagement, similar to the measure of extracurricular arts activities. Data were provided by the reading or mathematics/science teacher of each participant, meaning some responses varied within schools. We therefore calculated the mean number of arts classes reported within each school, averaged across all teacher reports.

Second, adequacy of arts facilities was derived from school administrator responses to ‘How adequate is each of the following school facilities [art room, music room, auditorium]?’, with response choices of 'do not have', 'never adequate', 'often not adequate', 'sometimes not adequate', and 'always adequate'. Responses were collapsed into 'do not have or inadequate' vs 'adequate’ and summed to create a score of the number of arts facilities that were adequate (0–3).

### Covariates

We included a range of sociodemographic factors and other covariates. Variables were largely defined by ECLS measures and were recategorized only when necessary due to small group sizes.

The individual-level covariates were: (1) gender (male vs female), (2) first-language (English vs Non-English) (3) ethnicity (White [non-Hispanic] vs Black or African American [non-Hispanic] vs Hispanic vs Other ethnicity [including Asian, Native Hawaiian, Other Pacific Islander, American Indian, Alaska Native, more than one race), (4) parental education indicating highest attainment of either parent (up to high school vs high school/vocational vs some college vs undergraduate vs postgraduate), (5) home location (city vs suburb/large town vs small town/rural), (6) family structure (married vs unmarried [separated/divorced/widowed] vs never married), (7) household income in the past year (quartiles), (8) family use of food stamps in past year (yes vs no), (9) student eligibility for reduced or free school meals (yes vs no),

The school-level covariates were: (1) type of school (private vs public), (2) percentage of students from ethnic minority groups (< 10% vs 10–25% vs 25–50% vs 50–75% vs > 75%), (3) overcrowding at the school (yes vs no), (4) school location (city vs suburb/large town vs small town/rural), and (5) school area safety in 5th grade (a summed score of the number of issues reported as a problem by the school administrator in the school area: racial tensions, unkempt area [garbage, litter, or broken glass in the streets, sidewalks, or yards], substance use [selling or using drugs or excessive drinking in public], gangs, heavy traffic, violent crime [e.g., drive-by shootings], vacant houses/buildings, and crime).

### Statistical analysis

Regression models were used to examine associations between extracurricular arts activities and school-based arts engagement in 5th grade with externalising behaviours in 8th grade. Ordinary least squares regression was used for both the individual-level and school-level analyses. Individual-level analyses were weighted according to age, ethnicity, education, and state in the US population to account for unequal sampling and were clustered by school. All models were initially unadjusted and then adjusted for covariates. Multiple imputation by chained equations (MICE) was conducted to address missing data in predictors, resulting in 50 imputed datasets for a final individual-level sample of 8315 and school-level sample of 357. All analyses were performed, and figures generated, using Stata 16^39^.

## Results

### Individual-level associations of 5th grade extracurricular arts activities and school-based arts engagement with 8th grade externalising behaviours

The 8315 participants in the individual-level sample had a mean age of 11.2 years (standard error [SE] = 0.01) in 5th grade, and 14.3 years (SE = 0.01) in 8th grade. In 5th grade, young people had participated in an average of 0.75 (SE = 0.02) extracurricular arts activities in the past year (Table 1). Just over half (52.5%) of the sample had not participated in any extracurricular arts activities in the past year. Schools offered a mean of 1.8 (SE = 0.03) arts-based classes at least weekly (range 0–6) and had an average of 1.7 (SE = 0.03) adequate arts facilities (range 0–3). Parents reported a mean of 3.6 (SE = 0.06) externalising behaviours when children were in 8th grade.

**Table 1:** Descriptive statistics for predictor variables in the individual-level analyses (n = 8315).

	Mean (SE)
Extracurricular arts activities	0.75 (0.02)
Number of art classes	1.82 (0.03)
Adequate arts facilities	1.72 (0.03)

	Percentage
Female	47.8
First language English	87.1
Ethnicity	
White	57.3
Black or African American	17.1
Hispanic	18.4
Other ethnicity [incl. AS, NH/OPI, AI/AN]	7.1
Parent education	
Up to high school	11.0
High school/vocational	30.4
Some college	27.2
Undergrad	18.0
Postgraduate and above	13.4
Location	
City	36.2
Suburb/large town	42.4
Small town/rural	21.4
Family structure	
Married	69.6
Unmarried	19.5
Never married	10.9
Income	
Quartile 1	32.1
Quartile 2	39.7
Quartile 3	13.1
Quartile 4	15.1
Received food stamps	14.8
Received free/reduced school meals	39.5

*AS* Asian, *NH/OPI* Native Hawaiian or Other Pacific Islander, *AI/AN* American Indian or Alaska Native, *SE* standard error. Results based on 50 multiply imputed datasets.

**Figure 1:**
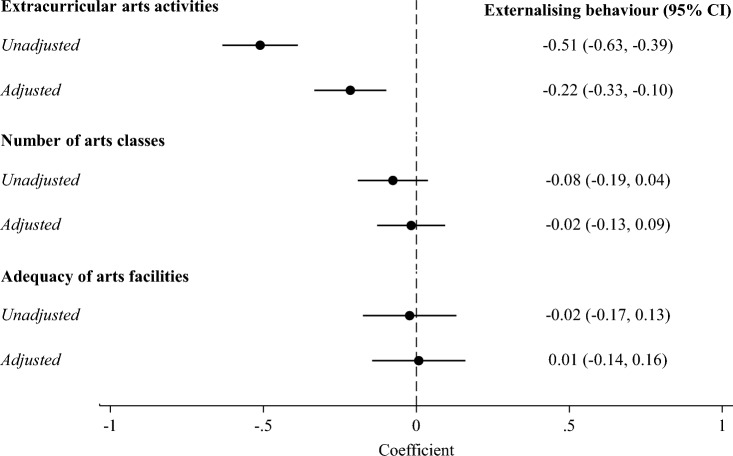
Individual-level associations of extracurricular and school-based arts engagement in 5th grade with externalising behaviours in 8th grade. Circles represent coefficients, which show the difference in externalising behaviours (ranging from 0 to 20) for each one-unit increase in arts engagement and are from ordinary least squares regression models in 50 imputed datasets. Horizontal lines show 95% confidence intervals. Number of extracurricular arts activities engaged in over the past 12 months ranged from 0 to 4, number of school arts classes offered 1–2 times weekly or more ranged from 0 to 6, and adequacy of school arts facilities ranged from 0 to 3. Models were adjusted for gender, first language, ethnicity, parental education, location, family structure, household income, use of food stamps, and eligibility for free/reduced school meals.

In the unadjusted model, participation in a greater number of extracurricular arts activities in 5th grade was associated with decreased individual-level externalising behaviours in 8th grade (regression coefficient [coef] − 0.51: 95% confidence interval [CI] − 0.63 to − 0.39; Fig. 1). This association was attenuated but remained significant in the adjusted model (coef − 0.22: 95% CI − 0.33 to − 0.10). Covariates associated with the largest reductions in externalising behaviours were those related to socioeconomic status, particularly higher parental education, ethnicity (Hispanic), and not qualifying for free school meals. There were no associations in either the unadjusted or adjusted models of arts classes or adequacy of arts facilities in 5th grade with individual-level externalising behaviours in 8th grade (full results available in Supplementary Table S2).

### School-level associations of 5th grade school-based arts engagement with 8th grade externalising behaviours

Of the 357 schools included in the sample, 57.1% were private, 35.7% had < 10% ethnic minority students, and 41.7% were in cities (Table 2). Teachers reported a mean of 2.0 (SE 0.07) arts classes per week (range 0–6) and 1.7 (SE 0.05) adequate arts facilities (range 0–3). School administrators reported a mean score of 5.3 (SE 0.2) on the externalising behaviours index (range 0–21).

**Table 2:** Descriptive statistics for predictor variables in the school-level analyses (n = 357).

	Mean (SE)
Number of art classes	2.03 (0.07)
Adequate art facilities	1.65 (0.05)
School area safety	1.71 (0.13)

	Percentage
School type	
Private	57.1
Public	42.9
Percent minority ethnic students	
< 10%	35.7
10–25%	18.3
25–50%	14.3
50–75%	7.3
> 75%	24.4
School overcrowded	28.2
School location	
City	41.7
Suburb/large town	34.3
Small town/rural	24.0

*SE* standard error. Results based on 50 multiply imputed datasets.

**Figure 2:**
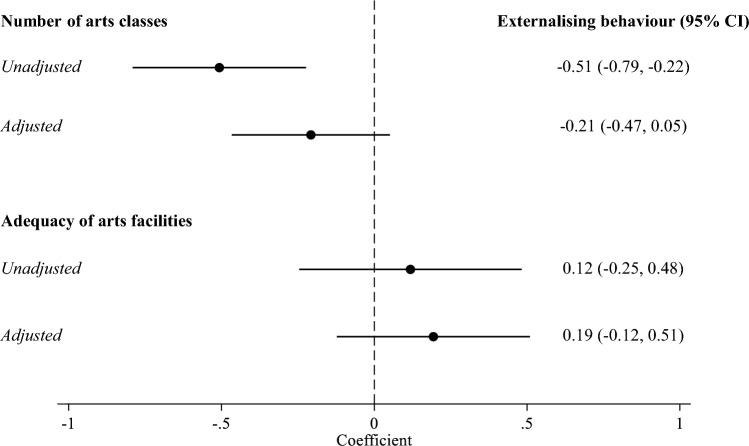
School-level associations of arts classes and adequacy of arts facilities in 5th grade with externalising behaviours in 8th grade. Circles represent coefficients, which show the difference in school externalising behaviour index (ranging from 0 to 21) for each one-unit increase in arts engagement and are from ordinary least squares regression models in 50 imputed datasets. Horizontal lines show 95% confidence intervals. Number of school arts classes offered 1–2 times weekly or more ranged from 0 to 6, and adequacy of school arts facilities ranged from 0 to 3. Models were adjusted for school-level covariates (school type, percentage of students from ethnic minority groups, school overcrowding, location, area safety).

In the unadjusted model, schools offering a greater number of weekly arts classes in 5th grade had fewer externalising behaviours in 8th grade (coef − 0.51: 95% CI − 0.79 to − 0.22; Fig. 2). However, this association was attenuated after adjusting for sociodemographic factors (coef − 0.21: 95% CI − 0.47 to 0.05). Public schools, schools with > 75% ethnic minority students, and those in less safe areas had more externalising behaviours (full results available in Supplementary Table S3).

There were no associations in either the unadjusted or adjusted models between adequacy of arts facilities in 5th grade with externalising behaviours in 8th grade at the school level (Fig. 2). However, as with provision of arts classes, public schools, schools with a larger proportion of ethnic minority students, and those in less safe areas had more externalising behaviours (Table S3).

## Discussion

This study examined whether extracurricular arts activities and school-based arts engagement in 5th grade were associated with reduced risk of externalising behaviours in 8th grade in a sample of US children and schools. In the individual-level analyses, we found that extracurricular arts activities in 5th grade (e.g., dance, music, or arts lessons or performing arts programs) were associated with decreased externalising behaviours reported by parents in 8th grade. We did not, however, find evidence of an association between school-based arts classes or adequacy of school arts facilities and later individual-level externalising behaviours. In our school-level analyses, more weekly arts classes were associated with decreased school administrator-reported externalising behaviours in 8th grade, although this association was attenuated following adjustment for school sociodemographic characteristics. There was no evidence of an association of adequacy of arts facilities with school-level externalising behaviours.

There has been little research into the use of arts as a risk reduction strategy for externalising behaviours (as opposed to as an intervention). Our findings show that engagement in extracurricular arts activities is associated with reduced risk of subsequent externalising behaviours. It is notable that the individual-level association was still present after adjustment for a range of demographic and socioeconomic covariates. Previous research has found strong associations between socioeconomic position, arts engagement^31^, and externalising behaviours^33–36^. Our findings align with the little longitudinal evidence available in this area and further support the theory that arts may be an effective risk reduction strategy^23,26^. There are numerous well-known benefits of arts engagement that may contribute to the reduction of externalising behaviours, which can occur on the individual, group, and/or societal level^40^. Improved emotion regulation and provision of supportive coping skills though art^41,42^ may help offset emotion dysregulation and negative behaviours such as aggression and impulsivity^14,43^. Skill development can lead to feelings of accomplishment, subsequently improving self-esteem and self-worth^44,45^. Further, social interaction and cohesion in art activities may be beneficial in increasing social competence and reducing antisocial and disruptive behaviour^8,15,27^.

In this study, the lack of individual-level associations between the school-based arts measures and externalising behaviours was unexpected. Although previous longitudinal research in this area has also been conducted inside schools, it has examined teacher rated creativity using free-writing, storytelling, handwork, painting, drawing, and drama^23^. This may suggest that it is the level of engagement a child has with the arts (i.e., actively creating or skill development) that is driving the associations with externalising behaviours, as opposed to purely exposure. In the current study, school-based arts exposures measured only opportunities for engagement (i.e., classes offered, facilities available), as no data were available on level of engagement with these resources. Availability of the arts alone may not reduce the risk of externalising behaviours. In line with this proposal, arts interventions often focus on engaging young people in creative expression. For example, a creative arts intervention was associated with reduced externalising behaviours in a sample of adolescents from refugee backgrounds^21^, while music-making was beneficial in reducing externalising behaviours in adolescents involved in the criminal justice system^27,28^. Interventions using drama^24^ and visual arts^25^ have been effective in the reduction of bullying in the classroom. Another longitudinal study found (parental rating of) arts abilities to be associated with decreased risk of externalising behaviours^26^. Future studies should further examine the differences between previous research and our findings, investigating the role of *actively* engaging in the arts compared to having *opportunities* to engage in the arts as potential risk reducing factors for externalising behaviours.

It is also of note that we did not find school-level associations for number of arts classes or adequacy of facilities. For the former, associations were present until we adjusted for socioeconomic factors affecting the schools. Public schools, for example, had fewer arts classes and more externalising behaviours. This suggests that simply offering more arts opportunities or improving facilities would be insufficient as a strategy to decrease externalising behaviours within schools. Several studies have examined how altering the school's physical environment (i.e., lighting, air quality, heating) may affect the student experience, however the results are mixed and largely focus on academic outcomes rather than behaviour^50^. Therefore, strategies to improve the student experience and behaviour should consider supporting extracurricular activities moving forward, given their associations with behavioural outcomes. However, it is possible that measurement error may have obscured potential associations. Teachers reported the number of arts classes and school administrators provided data on adequacy of arts facilities and externalising behaviours. School employees may have had positive response biases when rating school resources and interpretations of the word ‘adequate’ could vary according to the sociodemographic characteristics of the school. Although adjusting for socioeconomic covariates should have accounted for some of these differences, they might still have masked any association with subsequent externalising behaviours. More objective measures of the adequacy of school arts resources should be included in the future studies. Further, ECLS did not include a validated scale of externalising behaviours at the school level, so we created an index including a range of behavioural outcomes. We were therefore unable to directly compare individual and school-level results.

There are a number of strengths to the current study. ECLS is a large nationally representative cohort study with rich sociodemographic data, meaning we were able to adjust for a range of potential confounders^37^. We included reports from parents, teachers, and administrators and therefore reduced the likelihood of reporting bias by collecting similar information across several sources. We used a validated measure of externalising behaviours in the individual-level analyses (the SDQ)^38^. However, there are also several limitations to consider. The SDQ was parent-reported which may be subject to reporting bias, as parents may be more likely to report externalising behaviours compared to self-report SDQ assessments^51^. The wording of questions on individual-level extracurricular activities made it unclear whether these activities occurred independent of school or within the school setting (e.g., after hours school club). Therefore, it is difficult to fully disentangle school associated activities with those occurring outside school. Similarly, we were limited by the demographic categories available in the ECLS dataset. For example, gender was only reported as male vs female and language spoken at home as English vs non-English. Future cohort studies should endeavour to collect more detailed demographic data to ensure all individuals are equally and accurately represented. Additionally, while the ECLS weights meant that the individual-level data included in this study was nationally representative, the school-level data was not. There may therefore have been selection bias in the schools included in this study. Our school-level findings should thus be replicated in a nationally representative sample of schools in the US. However, the study sampling strategy should have mitigated some biases, selecting schools by geographic region and school type and, within each category, sorting schools to ensure sample representation of other characteristics^37^.

Given that this was an observational study, it is unclear if the association between art engagement and externalising behaviour is causal. Future research should use advanced methods for causal inference, alongside intervention studies, to establish whether arts engagement causally influences externalising behaviours. Adjustment for previous externalising behaviours should also be a priority. These data were not available in this study, meaning the findings could be a result of reverse causality. Additionally, we have provided preliminary evidence that parent-reported extracurricular arts engagement was associated with externalising behaviours three years later. This leads to several further questions that require investigation. First, it is unclear to what extent participation in these extracurricular arts activities is driven by self-motivation or external factors (e.g. peer pressure, teacher/parental encouragement). This is likely to influence young people’s motivation and level and quality of engagement, which may then alter the association with externalising behaviours. Second, whether longitudinal changes in arts engagement are associated with changes in externalising behaviours should be explored, particularly as young people gain more autonomy over their extracurricular activities with age. Third, due to data constraints, we were not able to examine the frequency of engagement. We would expect more frequent arts engagement to be associated with larger reductions in externalising behaviours; this dose–response relationship should be investigated further. Finally, whether specific forms of arts engagement differentially influence externalising behaviours remains to be explored^46^. Different art forms require varied skills and have different goals^47^ and there is evidence that participation in acting (compared to visual arts or music) may have contrasting effects on social and cognitive skills^48^, although engagement in visual arts, performing arts, and literature have all been linked to prosocial behaviour^49^.

Furthermore, it is critical to note that neurological differences are often at the root of the behaviours known as externalising behaviours, and these behaviours may in some way be protective against the detrimental health consequences of early life adversity and neurological differences^52^. We did not have such variables available to include in our analyses, but future research is needed to consider these differences as, when they are overlooked, problematic behaviours and their consequences can escalate and create a cycle of perpetuation. Additionally, when behaviours related to neurodivergence are linked to school discipline and criminality, factors such as structural determinism, racism, and social disenfranchisement must be considered^53–55^. So, although we took care to include a rich panel of demographic and socioeconomic confounders in our analyses, we may have omitted important further confounders that were not available in the dataset. Finally, we have followed current conventions for describing externalising behaviours, but we recognise that it may be useful to use less 'problematic' and 'deficit' heavy language when referring to differences in neurodivergence^55,56^.

Our results suggest that extracurricular arts activities (e.g., dance, music, or arts lessons or performing arts programs) may be effective in reducing the risk for externalising behaviours. However, the findings also caution against simply increasing facilities in schools as a way to tackle behavioural issues. Instead, it appears that more targeted provision of extracurricular classes, in particular aiming to reach those who are less likely to engage outside of school time, is important. Future research is recommended to extend our findings, which highlight important directions for future work. Overall, the benefits of arts engagement are well established, and many of these benefits parallel the mechanisms underlying externalising behaviours. Art has previously been used as an intervention. However, our evidence suggests it may also be an efficient tool in reducing the risk of externalising behaviours in the first instance through more general extracurricular activity provision.

### Supplementary Information

Supplementary Tables.

## Data Availability

The datasets analysed during the current study are available in the National Center for Education Statistics repository, https://nces.ed.gov/ecls/dataproducts.asp#K-8.
