# Supplementary material for: Associations between extracurricular arts activities, school-based arts engagement, and subsequent externalising behaviours in the Early Childhood Longitudinal Study
Source: Sci Rep. 2023 Aug 24;13:13840. doi: 10.1038/s41598-023-39925-6 (PMC10449872; doi:10.1038/s41598-023-39925-6)
Supplement: Supplementary file 1 — Supplementary Tables. [file 41598_2023_39925_MOESM1_ESM.docx]

**Supplementary Material**

**Table S1.** Proportion of missing data in predictor variables.

|  | Proportion missing |
| --- | --- |
| **Individual-level analyses (n=8,315 students)** |  |
| Extracurricular arts activities | 0.01% |
| Number art classes | 14.3% |
| Adequate art facilities | 10.8% |
| Gender | - |
| First language | 3.5% |
| Ethnicity | 0.1% |
| Parent education | 2.9% |
| Location | 0.6% |
| Family structure | 0.1% |
| Income | 6.3% |
| Food stamps | 0.1% |
| Free/reduced school meals | 0.01% |
| **School-level analyses (n=357 schools)** |  |
| Number art classes | 9.0% |
| Adequate art facilities | 7.3% |
| Type | - |
| Percent ethnic minority students | 0.3% |
| Overcrowded | 5.3% |
| Location | 7.3% |
| School area safety | 26.3% |

**Table S2.** Individual-level associations of extracurricular and school-based arts engagement in 5^th^ grade with externalising behaviours in 8^th^ grade.

|  | **Externalising behaviours** | | | | | | | | | | | | | |
| --- | --- | --- | --- | --- | --- | --- | --- | --- | --- | --- | --- | --- | --- | --- |
|  | **Extracurricular arts activities** | | | |  | **Number of arts classes** | | | |  | **Adequacy of arts facilities** | | | |
|  | *Coef.* | *95% CI* | | *P* |  | *Coef.* | *95% CI* | | *P* |  | *Coef.* | *95% CI* | | *P* |
| **Unadjusted** |  |  |  |  |  |  |  |  |  |  |  |  |  |  |
| Arts exposure | **-0.51** | **-0.63** | **-0.39** | **<0.001** |  | -0.08 | -0.19 | 0.04 | 0.189 |  | -0.02 | -0.17 | 0.13 | 0.777 |
| **Adjusted** |  |  |  |  |  |  |  |  |  |  |  |  |  |  |
| Arts exposure | **-0.22** | **-0.33** | **-0.10** | **<0.001** |  | -0.02 | -0.13 | 0.09 | 0.765 |  | 0.01 | -0.14 | 0.16 | 0.918 |
| **Gender** (Male) | - |  |  |  |  | - |  |  |  |  | - |  |  |  |
| Female | **-1.02** | **-1.26** | **-0.80** | **<0.001** |  | **-1.13** | **-1.37** | **-0.89** | **<0.001** |  | **-1.13** | **-1.37** | **-0.89** | **<0.001** |
| **First language** (English) | - |  |  |  |  | - |  |  |  |  | - |  |  |  |
| Non-English | **-0.53** | **-0.91** | **-0.15** | **0.007** |  | **-0.58** | **-0.96** | **-0.19** | **0.003** |  | **-0.57** | **-0.96** | **-0.19** | **0.003** |
| **Ethnicity** (White) | - |  |  |  |  | - |  |  |  |  | - |  |  |  |
| Black or African American | -0.31 | -0.75 | 0.14 | 0.177 |  | -0.36 | -0.81 | 0.09 | 0.115 |  | -0.36 | -0.81 | 0.14 | 0.115 |
| Hispanic | **-0.66** | **-0.98** | **-0.34** | **<0.001** |  | **-0.65** | **-0.97** | **-0.33** | **<0.001** |  | **-0.65** | **-0.98** | **-0.32** | **<0.001** |
| Other ethnicity [incl. AS, NH/OPI, AI/AN] | **-0.55** | **-0.91** | **-0.18** | **0.003** |  | **-0.56** | **-0.93** | **-0.20** | **0.003** |  | **-0.56** | **-0.94** | **-0.19** | **0.003** |
| **Parent education** (Up to high school) | - |  |  |  |  | - |  |  |  |  | - |  |  |  |
| High school/vocational | -0.39 | -0.92 | 0.14 | 0.146 |  | -0.43 | -0.95 | 0.10 | 0.109 |  | -0.43 | -0.96 | 0.09 | 0.107 |
| Some college | **-0.68** | **-1.21** | **-0.14** | **0.013** |  | **-0.73** | **-1.27** | **-0.20** | **0.007** |  | **-0.74** | **-1.27** | **-0.21** | **0.006** |
| Undergrad | **-1.02** | **-1.56** | **-0.48** | **<0.001** |  | **-1.11** | **-1.64** | **-0.57** | **<0.001** |  | **-1.11** | **-1.65** | **-0.58** | **<0.001** |
| Postgraduate and above | **-0.97** | **-1.52** | **-0.42** | **0.001** |  | **-1.12** | **-1.66** | **-0.57** | **<0.001** |  | **-1.12** | **-1.67** | **-0.58** | **<0.001** |
| **Location** (City) | - |  |  |  |  | - |  |  |  |  | - |  |  |  |
| Suburb/large town | -0.05 | -0.32 | 0.22 | 0.727 |  | -0.05 | -0.32 | 0.22 | 0.714 |  | -0.05 | -0.32 | 0.22 | 0.712 |
| Small town/rural | -0.09 | -0.41 | 0.23 | 0.594 |  | -0.08 | -0.40 | 0.24 | 0.625 |  | -0.08 | -0.40 | 0.24 | 0.632 |
| **Family structure** (Married) | - |  |  |  |  | - |  |  |  |  | - |  |  |  |
| Unmarried | **0.56** | **0.16** | **0.95** | **0.005** |  | **0.58** | **0.18** | **0.97** | **0.004** |  | **0.58** | **0.19** | **0.97** | **0.004** |
| Never married | -0.06 | -0.55 | 0.43 | 0.808 |  | -0.03 | -0.52 | 0.46 | 0.903 |  | -0.03 | -0.52 | 0.46 | 0.905 |
| **Household income** (Quartile 1) | - |  |  |  |  | - |  |  |  |  | - |  |  |  |
| Quartile 2 | -0.29 | -0.68 | 0.10 | 0.140 |  | -0.30 | -0.69 | 0.09 | 0.137 |  | -0.30 | -0.69 | 0.09 | 0.134 |
| Quartile 3 | 0.07 | -0.65 | 0.78 | 0.856 |  | 0.05 | -0.67 | 0.77 | 0.894 |  | 0.05 | -0.67 | 0.77 | 0.900 |
| Quartile 4 | -0.35 | -0.86 | 0.16 | 0.178 |  | -0.39 | -0.90 | 0.13 | 0.139 |  | -0.39 | -0.90 | 0.12 | 0.137 |
| **Food stamps** (No) | - |  |  |  |  | - |  |  |  |  | - |  |  |  |
| Yes | **0.57** | **0.12** | **1.02** | **0.012** |  | **0.58** | **0.13** | **1.03** | **0.011** |  | **0.58** | **0.13** | **1.03** | **0.012** |
| **Free/reduced school meals** (No) | - |  |  |  |  | - |  |  |  |  | - |  |  |  |
| Yes | **0.63** | **0.2** | **1.03** | **0.002** |  | **0.63** | **0.23** | **1.04** | **0.002** |  | **0.64** | **0.23** | **1.04** | **0.002** |

*Note.* Extracurricular arts activities is a continuous score (0-4) of the number of extracurricular arts activities engaged in over the past 12 months. Number of arts classes is a continuous score of the number of arts classes offered 1-2 weekly or more (0-6). Adequacy of arts facilities is a continuous score (0-3). Outcome variable is continuous score (0-20) of SDQ externalising behaviours. AS= Asian, NH/OPI= Native Hawaiian or Other Pacific Islander, AI/AN= American Indian or Alaska Native. Dashes indicate the reference category. Bold text indicates p<0.05.

**Table S3.** School-level associations of arts classes and adequate arts facilities in 5^th^ grade with externalising behaviours in 8^th^ grade.

|  | **Externalising behaviours index** | | | | | | | | | |
| --- | --- | --- | --- | --- | --- | --- | --- | --- | --- | --- |
|  |  | **Number of arts classes** | | | |  | **Adequacy of arts facilities** | | | |
|  |  | *Coef.* | *95% CI* | | *P* |  | *Coef.* | *95% CI* | | *P* |
| **Unadjusted** |  |  |  |  |  |  |  |  |  |  |
| Arts exposure |  | **-0.51** | **-0.79** | **-0.22** | **0.001** |  | 0.12 | -0.25 | 0.48 | 0.522 |
| **Adjusted** |  |  |  |  |  |  |  |  |  |  |
| Arts exposure |  | -0.21 | -0.47 | 0.05 | 0.116 |  | 0.19 | -0.12 | 0.51 | 0.229 |
| **Type** (Private) |  | **-** |  |  |  |  | **-** |  |  |  |
| Public |  | **2.87** | **2.15** | **3.60** | **<0.001** |  | **2.98** | **2.27** | **3.69** | **<0.001** |
| **Percent ethnic minority** (<10%) |  | **-** |  |  |  |  | **-** |  |  |  |
| 10-25% |  | 0.85 | -0.01 | 1.71 | 0.052 |  | 0.84 | -0.02 | 1.70 | 0.056 |
| 25-50% |  | 0.43 | 0.50 | 1.36 | 0.367 |  | 0.44 | -0.49 | 1.38 | 0.349 |
| 50-75% |  | 1.06 | -0.15 | 2.28 | 0.086 |  | 1.04 | -0.17 | 2.26 | 0.092 |
| >75% |  | **1.37** | **0.40** | **2.34** | **0.006** |  | **1.42** | **0.46** | **2.39** | **0.004** |
| **School is overcrowded** (No) |  | **-** |  |  |  |  | **-** |  |  |  |
| Yes |  | 0.05 | -0.64 | 0.74 | 0.890 |  | 0.05 | -0.64 | 0.74 | 0.884 |
| **Location** (City) |  | **-** |  |  |  |  | **-** |  |  |  |
| Suburb/large town |  | -0.28 | -1.01 | 0.44 | 0.440 |  | -0.31 | -1.03 | 0.41 | 0.401 |
| Small town/rural |  | -0.59 | -1.51 | 0.34 | 0.211 |  | -0.50 | -1.43 | 0.42 | 0.286 |
| **School area safety** |  | **0.19** | **0.01** | **0.38** | **0.038** |  | **0.19** | **0.01** | **0.38** | **0.043** |

*Note.* Number of arts classes variable is a continuous score of the number of arts classes offered 1-2 weekly or more (0-6). Adequacy of arts facilities is a continuous score (0-3). Outcome variable is a continuous index of the number of externalising behaviours (0-7) in the school. Dashes indicate the reference category. Bold text indicates p<0.05.
